# Cytochrome *bd*-Dependent Bioenergetics and Antinitrosative Defenses in *Salmonella* Pathogenesis

**DOI:** 10.1128/mBio.02052-16

**Published:** 2016-12-20

**Authors:** Jessica Jones-Carson, Maroof Husain, Lin Liu, David J. Orlicky, Andrés Vázquez-Torres

**Affiliations:** a Division of Infectious Diseases, University of Colorado School of Medicine, Aurora, Colorado, USA; b Department of Immunology and Microbiology, University of Colorado School of Medicine, Aurora, Colorado, USA; c Department of Pathology, University of Colorado School of Medicine, Aurora, Colorado, USA; d Veterans Affairs Eastern Colorado Health Care System, Denver, Colorado, USA

## Abstract

In the course of an infection, *Salmonella enterica* occupies diverse anatomical sites with various concentrations of oxygen (O_2_) and nitric oxide (NO). These diatomic gases compete for binding to catalytic metal groups of quinol oxidases. *Enterobacteriaceae* express two evolutionarily distinct classes of quinol oxidases that differ in affinity for O_2_ and NO as well as stoichiometry of H^+^ translocated across the cytoplasmic membrane. The investigations presented here show that the dual function of bacterial cytochrome *bd* in bioenergetics and antinitrosative defense enhances *Salmonella* virulence. The high affinity of cytochrome *bd* for O_2_ optimizes respiratory rates in hypoxic cultures, and thus, this quinol oxidase maximizes bacterial growth under O_2_-limiting conditions. Our investigations also indicate that cytochrome *bd*, rather than cytochrome *bo*, is an intrinsic component of the adaptive antinitrosative toolbox of *Salmonella*. Accordingly, induction of cytochrome *bd* helps *Salmonella* grow and respire in the presence of inhibitory NO. The combined antinitrosative defenses of cytochrome *bd* and the flavohemoglobin Hmp account for a great part of the adaptations that help *Salmonella* recover from the antimicrobial activity of NO. Moreover, the antinitrosative defenses of cytochrome *bd* and flavohemoglobin Hmp synergize to promote *Salmonella* growth in systemic tissues. Collectively, our investigations indicate that cytochrome *bd* is a critical means by which *Salmonella* resists the nitrosative stress that is engendered in the innate response of mammalian hosts while it concomitantly allows for proper O_2_ utilization in tissue hypoxia.

## IMPORTANCE

It is becoming quite apparent that metabolism is critically important to the virulence potential of pathogenic microorganisms. Bacterial cells use a variety of terminal electron acceptors to power electron transport chains and metabolic processes. Of all the electron acceptors available to bacteria, utilization of O_2_ yields the most energy while diversifying the type of substrates that a pathogen can use. Recent investigations have demonstrated important roles for *bd*-type quinol oxidases with high affinity for O_2_ in bacterial pathogenesis. The investigations presented here have revealed that cytochrome *bd* potentiates virulence of a clinically relevant bacterial pathogen by fueling bioenergetics of prokaryotic cells while protecting the respiratory chain against NO toxicity. The adaptive antinitrosative defenses afforded by cytochrome *bd* synergize with other NO-detoxifying systems to preserve cellular bioenergetics, thereby promoting bacterial virulence in tissue hypoxia.

## INTRODUCTION

*Salmonella enterica* serovar Typhimurium is a common cause of nontyphoidal salmonellosis in humans and domestic animals. In most healthy individuals, nontyphoidal *Salmonella* infections acquired from diverse vertebrate hosts via the fecal/oral route present as self-limiting gastroenteritis. Nonetheless, in immunocompromised people bearing defects in CD4^+^ T cell immunity or gamma interferon (IFN-γ) signaling, diverse strains of nontyphoidal *Salmonella* can cause life-threatening extraintestinal infections (1–3). *Salmonella* suffers the cytotoxicity of cationic peptides, as well as reactive oxygen and nitrogen species that are generated in the host response of vertebrate animals and humans. Nitric oxide (NO) is one of the most studied anti-*Salmonella* effectors of the innate response in mammalian cells (4). The oxidation of the guanidino group of l-arginine by the enzymatic activity of NO synthases generates NO and l-citrulline (5). Reactions of NO with superoxide, molecular oxygen (O_2_), iron, or low-molecular-weight thiols produce an amalgam of antimicrobial reactive nitrogen species that include peroxynitrite, nitrogen dioxide, dinitrogen trioxide, and S-nitrosothiols. A collection of reactive nitrogen species can independently be generated upon the condensation of two molecules of acidified nitrite in the stomach and phagosomal lumen of macrophages (6–10). NO and its oxidative and nitrosative congeners exert antimicrobial activity against diverse eukaryotic and prokaryotic organisms. Cytochrome *bd*, DNA, lipoamide-dependent lipoamide dehydrogenase, and the regulatory proteins DksA and SsrB are some of the few biomolecules known to be modified in *Salmonella* undergoing nitrosative stress (11–15).

Despite the potent antimicrobial activity that NO can exert against *Salmonella*, this intracellular pathogen tolerates remarkably well the nitrosative stress engendered in the innate host response (8, 16). Diverse antinitrosative defenses help *Salmonella* cope with NO and its oxidative by-products. For example, the low-molecular-weight thiols homocysteine and glutathione scavenge reactive nitrogen species, whereas the denitrosylase activity of the flavohemoglobin Hmp detoxifies NO to nitrate (NO_3_^−^) (17–19). The combined actions of low-molecular-weight thiols and Hmp protect *Salmonella* against the nitrosative stress engendered in the innate host response of human and murine macrophages (20–24). Not only does *Salmonella* tolerate NO-mediated host defenses, but these pathogens can also take advantage of the redox properties of nitrogen oxides to colonize the gastrointestinal tract. For example, terminal cytochromes such as nitrate reductases energize cytoplasmic membranes by utilizing NO oxidative products as terminal electron acceptors (25).

Reduction of O_2_ to water is the canonical function of aerobic terminal cytochromes of the electron transport chain, a process that generates an electrochemical gradient across cytoplasmic membranes and powers transport systems and ATP synthesis. *Salmonella* expresses two evolutionarily distinct classes of quinol oxidases. Cytochrome *bo*, a member of the cytochrome *c* oxidase family, is encoded in the *cyoABCD* operon, whereas cytochrome *bd* and cytochrome *bd*-II are encoded in the *cydAB* and *cyxAB* operons, respectively. Cytochrome *bd* and cytochrome *bd*-II contain heme *d* in place of the Cu_B_ atom that occupies the catalytic site of cytochrome *bo* (26). Although less efficient than cytochrome *bo*, cytochrome *bd* also participates in the bioenergetics of the bacterial cell (26). The expression of cytochrome *bd* in *S. enterica*, *Klebsiella pneumoniae*, *Mycobacterium tuberculosis*, *Shigella flexneri*, group B *Streptococcus*, *Listeria monocytogenes*, and *Bacteroides* suggests a possible role for this quinol oxidase in bacterial pathogenesis (27). Accordingly, cytochrome *bd* promotes gastrointestinal and systemic fitness of *Citrobacter rodentium* and *S. enterica* serovar Typhimurium, respectively (28–30).

Expression of cytochrome *bd* in *E. coli*, *Staphylococcus aureus*, *Bacillus subtilis*, and *M. tuberculosis* in response to NO and the nitrosylation of the heme *d* in cytochrome *bd* of *Salmonella* raise the interesting possibility that, in addition to fueling the bioenergetics of the cell, cytochrome *bd* may be part of the antinitrosative arsenal of several pathogenic microorganisms (11, 31–35). This idea is suggested further by the fact that Δ*cydAB E. coli* and Δ*fur Salmonella*, a strain that harbors low concentrations of cytochrome *bd*, are hypersusceptible to the bacteriostatic activity of chemically generated NO (16, 31, 36). However, given the copious antinitrosative defenses available to pathogenic bacteria (4), it remains uncertain whether the NO-detoxifying activity of cytochrome *bd* contributes to bacterial virulence. The following investigations have explored the extent to which cytochrome *bd* contributes to the antinitrosative defenses of *Salmonella* in culture and murine models of infection.

## RESULTS

### Contribution of quinol oxidases to *Salmonella* virulence.

**FIG 1:**
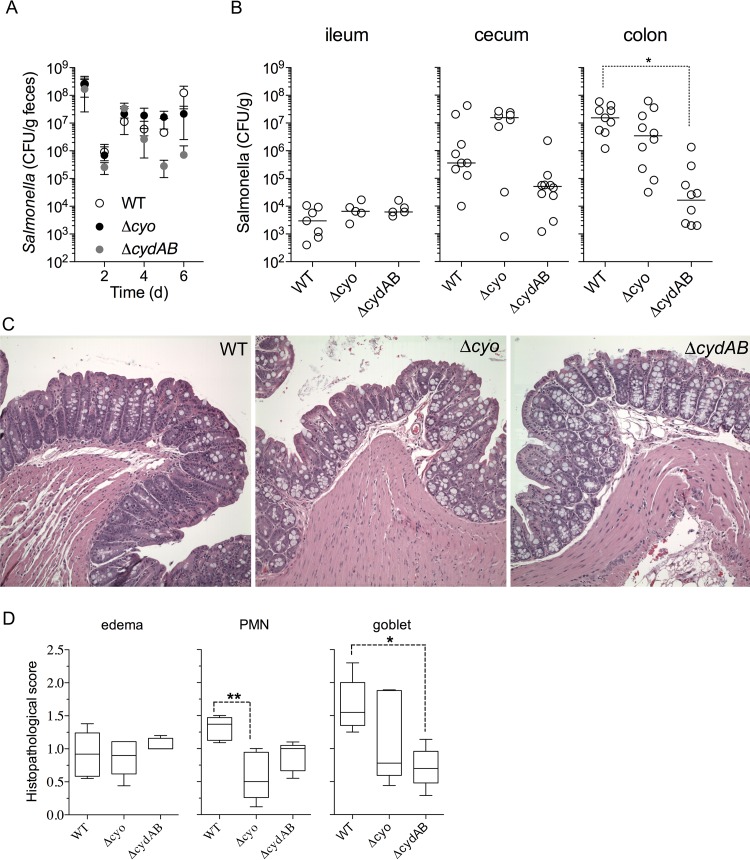
Quinol oxidases and colonization of the gastrointestinal tract by *Salmonella*. (A) Fecal shedding in streptomycin-treated C3H/HeN mice infected p.o. with wild-type (WT), Δ*cyoABCD,* or Δ*cydAB Salmonella*. (B) The *Salmonella* burden in small and large intestines was determined 3 days after infection. The solid line represents the median. (C) Histopathology of paraffin-embedded, hematoxylin-and-eosin-stained ceca isolated 3 days postinfection. Representative images (×200 magnification) were collected as described in Materials and Methods. (D) The severity of edema in submucosa, polymorphonuclear leukocyte (PMN) infiltration in mucosa, and depletion of goblet cells was scored according to the method described by Barthel et al. (52). The data are from 5 to 10 mice. *, *P* < 0.05; **, *P* < 0.01.

*Salmonella* expresses two major terminal quinol oxidases encoded within *cyoABCD* and *cydAB* operons. Compared to *cydAB*-encoded cytochrome *bd*, cytochrome *bo* has lower affinity for O_2_ and NO but greater capacity to translocate protons across the cytoplasmic membrane (27, 36). Given the pronounced differences between these two quinol oxidases, we compared the capacities of Δ*cyoABCD* and Δ*cydAB* mutants to colonize the gastrointestinal tract of streptomycin-treated C3H/HeN mice. Similar amounts of Δ*cyoABCD* and Δ*cydAB Salmonella* were shed in feces of C3H/HeN mice compared to control mice infected with an isogenic wild-type strain (Fig. 1A). With the exception of the Δ*cydAB* mutant, which was recovered in lower numbers in colon, Δ*cyoABCD*- and Δ*cydAB*-deficient *Salmonella* appeared to be as capable as wild-type bacteria in colonizing the small and large intestines of streptomycin-treated C3H/HeN mice 3 days after oral (p.o.) inoculation. All strains tested also induced similar levels of inflammation in ceca of infected animals as indicated by the presence of edema in the submucosa, infiltration of polymorphonuclear cells in the lamina propria, and depletion of goblet cells (Fig. 1C and D). Despite these similarities, some differences in histopathology were noted. For instance, ceca of Δ*cyoABCD Salmonella*-infected mice contained fewer polymorphonuclear cells than wild-type controls, whereas the ceca of mice infected with Δ*cydAB Salmonella* contained more goblet cells than controls infected with either wild-type or Δ*cyoABCD Salmonella*. Collectively, our investigations are consistent with recently published data that showed the apparent dispensability of cytochrome *bd* during colonization of the gut (28).

### Cytochrome *bd* contributes to *Salmonella* antinitrosative defenses.

**FIG 2:**
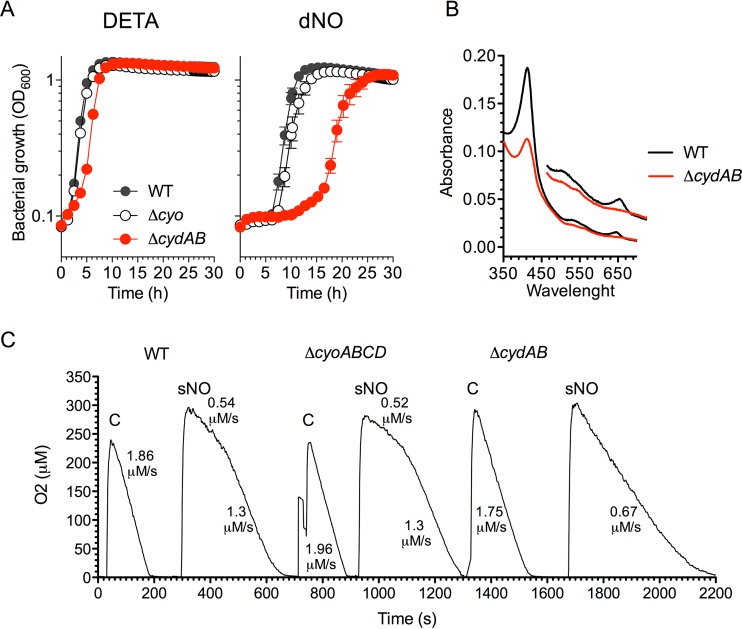
Contribution of quinol oxidases to the antinitrosative defenses of *Salmonella*. (A) Wild-type (WT), Δ*cyo,* or Δ*cydAB Salmonella* grown overnight in LB broth was diluted to 2 × 10^6^ CFU/ml in fresh LB broth. Where indicated, bacterial cultures were treated with 5 mM DETA or NO donor DETA NONOate (dNO). Bacterial growth was recorded using a Bioscreen C growth analyzer at 37°C with continuous shaking. Data represent the means ± standard errors of the means from 10 observations from two different experiments. (B) Absorption spectra of cytoplasmic membranes isolated from stationary-phase WT or Δ*cydAB Salmonella* grown in LB broth. The inset shows a detail of the 480- to 690-nm region. (C) Effect of NO on respiration. Stationary-phase *Salmonella* was grown to an OD_600_ of 0.5 in LB broth at 37°C in a shaker incubator. Bacterial cultures were diluted to an OD_600_ of 0.2, and O_2_ consumption was recorded over time. Prior to analysis, bacteria were treated with 50 μM spermine NONOate (sNO) for 1 min. Untreated controls (C) are shown for comparison. Data are representative of three independent experiments.

Previous work showed that Δ*fur Salmonella* is hypersusceptible to the bacteriostatic activity of NO generated chemically *in vitro* or enzymatically *in vivo* (16). Diminished 420-nm and 560-nm absorption peaks in cytoplasmic membranes of Δ*fur Salmonella* indicate that this mutant harbors reduced concentrations of both cytochrome *bo* and cytochrome *bd*. To investigate a possible role of these two terminal cytochromes in the antinitrosative defenses of *Salmonella*, we compared growth rates of Δ*cyoABCD* and Δ*cydAB* mutants deficient in cytochrome *bo* and cytochrome *bd*, respectively, in LB broth supplemented with diethylenetriamine (DETA) or the NO donor DETA NONOate (Fig. 2A). Wild-type and Δ*cyoABCD Salmonella* strains grew similarly in LB broth containing 5 mM DETA. The addition of 5 mM DETA NONOate, which is estimated to generate a constant flux of 5 μM NO for the duration of the experiment, inhibited the growth of wild-type and Δ*cyoABCD* bacteria to similar extents. These findings indicate that cytochrome *bo* does not constitute an important component of the antinitrosative arsenal of *Salmonella*. We also tested a Δ*cydAB* mutant whose cytoplasmic membranes lack the characteristic 650-nm absorption peak of heme *d* (Fig. 2B). In contrast to wild-type and Δ*cyoABCD* isogenic controls, Δ*cydAB Salmonella* consistently grew more slowly in LB broth, suggesting that the high affinity for O_2_ of cytochrome *bd* improves growth of *Salmonella* in hypoxic media. *Salmonella* bearing the Δ*cydAB* mutation was also hypersusceptible to the bacteriostatic activity of NO as suggested by the extended lag phase that followed DETA NONOate treatment. Cumulatively, these investigations indicate that cytochrome *bd*, rather than cytochrome *bo*, is an intrinsic constituent of the antinitrosative defenses of *Salmonella*.

### Cytochrome *bd* protects respiration against NO.

Since terminal cytochromes of the electron transport chain are some of the preferred targets of NO (4), we measured the rates of respiration in Δ*cyoABC* and Δ*cydAB Salmonella* exposed to spermine NONOate (Fig. 2C). Wild-type and Δ*cyoABCD Salmonella* strains grown to log phase in LB broth showed comparable respiratory activities. Addition of 50 μM NO donor spermine NONOate similarly repressed the respiratory activity of wild-type and Δ*cyoABCD Salmonella* (0.54 versus 0.52 μM O_2_/s, respectively). Respiratory rates improved in both wild-type and Δ*cyoABCD Salmonella* 3.5 min after treatment (1.3 μM/s) but did not reach those recorded in resting cells (~1.90 μM/s). The Δ*cydAB Salmonella* strain respired more slowly than unstimulated wild-type or Δ*cyoABCD* controls, strains that likely take advantage of the superior performance of cytochrome *bd* under hypoxia. Compared to wild-type bacteria, the respiratory activity of the Δ*cydAB* mutant was considerably more sensitive to the inhibitory effects of spermine NONOate. Furthermore, in contrast to wild-type and Δ*cyoABCD Salmonella*, the respiratory activity of Δ*cydAB Salmonella* did not seem to improve over time after the addition of spermine NONOate (0.67 μM/s). These findings indicate that cytochrome *bd*, but not cytochrome *bo*, affords protection to quinol oxidases against NO toxicity.

### Cytochrome *bd* and the flavohemoglobin Hmp lessen NO cytotoxicity.

**FIG 3:**
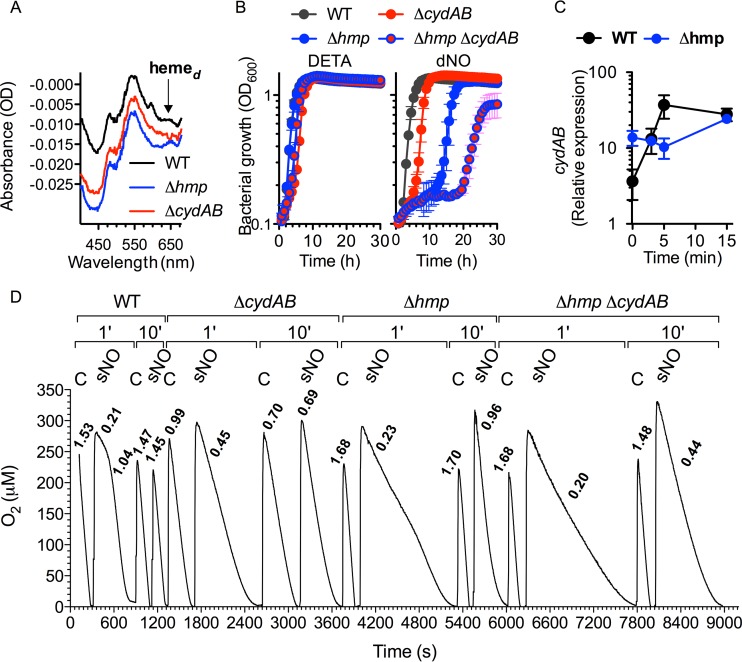
Synergy of Hmp and cytochrome *bd* in the antinitrosative defenses of *Salmonella*. (A) Differential whole-cell spectra of wild-type (WT) and mutant *Salmonella*. The arrow shows the absorption peak of heme *d*. (B) Growth of *Salmonella* diluted to 2 × 10^6^ CFU/ml in LB broth after treatment with 1 mM DETA or DETA NONOate (dNO). Data represent the means ± standard errors of the means from 10 observations from two independent experiments. (C) Expression of *cydA* in wild-type and Δ*hmp Salmonella* exposed to 50 μM spermine NONOate (sNO). Time after addition of NO (*P* < 0.0001) and bacterial strain (*P* = 0.0117) were found to statistically affect the expression of *cydA* as determined by two-way analysis of variance (*n* = 4 from two independent days). No *cydA* mRNA was detected in Δ*cydAB Salmonella* (not shown). (D) Effect of NO on respiration. Bacterial cells were prepared as described in the legend to Fig. 2. Where indicated, bacteria were treated with 50 μM sNO for 1 or 10 min prior to the analysis of respiration. Untreated controls (C) are shown for comparison. Data are representative of 3 independent experiments.

Previous work identified the flavohemoglobin Hmp as the main antinitrosative defense of *Salmonella* (22). Because our investigations indicate that cytochrome *bd* is part of the antinitrosative arsenal of *Salmonella*, we deemed it important to compare the relative contributions of Hmp and cytochrome *bd* to the antinitrosative defenses of *Salmonella*. Toward this end, a Δ*cydAB*::Km mutation was moved into Δ*hmp Salmonella* strain AV0468. Differential spectrophotometry of whole bacterial cells indicates that Δ*hmp Salmonella* expresses higher concentrations of cytochrome *bd* than wild-type controls as shown by the characteristic 650-nm heme *d* absorption peak (Fig. 3A). We initially tested the susceptibility of *hmp*-deficient *Salmonella* strains to 5 mM DETA NONOate; however, at this concentration the NO donor completely inhibited growth of both Δ*hmp* and Δ*hmp ΔcydAB*::Km *Salmonella*. Therefore, we tested lower concentrations of DETA NONOate and the DETA parent compound. The addition of 1 mM DETA did not have much of an effect on the growth of wild-type *Salmonella* (Fig. 3B). As noted above for Δ*cydAB Salmonella*, the Δ*hmp* Δ*cydAB* strain exhibited a slight but reproducible growth defect in LB broth. The addition of 1 mM DETA NONOate did not inhibit growth of wild-type *Salmonella*. However, 1 mM DETA NONOate extended in increasing order the lag phase of *ΔcydAB*, Δ*hmp*, and Δ*hmp ΔcydAB*::Km mutant *Salmonella* strains. Together, our investigations indicate that both Hmp and cytochrome *bd* protect *Salmonella* against NO, although quantitatively the flavohemoglobin appears to confer the greatest protection. Because Δ*hmp ΔcydAB*::Km mutant *Salmonella* was more susceptible to NO than *ΔcydAB* or Δ*hmp* isogenic strains, our investigations also indicate that Hmp and cytochrome *bd* independently add to the antinitrosative arsenal of *Salmonella*.

Transcriptional analysis showed that the *cydA* operon is induced in wild-type *Salmonella* shortly after the addition of 50 μM spermine NONOate (*P* < 0.0001) (Fig. 3C). Consistent with the spectroscopic analysis shown in Fig. 3A, Δ*hmp Salmonella* expressed higher levels of *cydA* than wild-type controls (*P* = 0.0117). Although delayed compared to wild-type controls, NO also induced *cydAB* expression in Δ*hmp Salmonella* (*P* < 0.0001). Cumulatively, these data indicate that cytochrome *bd* is part of the adaptive response of *Salmonella* to nitrosative stress.

### Cytochrome *bd* and Hmp independently protect the respiratory chain from the inhibitory activity of NO.

We next examined the rates of respiration of *Salmonella* strains deficient in Hmp and/or cytochrome *bd*. Wild-type *Salmonella* exposed for 1 min to 50 μM spermine NONOate exhibited reduced respiratory activity (Fig. 3D). As shown above, wild-type *Salmonella* recovered about two-thirds of its maximal respiratory activity a few minutes after NO treatment, consistent with the stimulation of a partial adaptive response. To test if *Salmonella* can fully adapt to NO, respiratory activity was independently measured 10 min after spermine NONOate treatment. Wild-type *Salmonella* completely recovered O_2_-consuming capacity 10 min after spermine NONOate treatment (1.47 versus 1.45 μM/s for nontreated and NO-treated specimens, respectively). As observed earlier (Fig. 2C), untreated Δ*cydAB Salmonella* exhibited lower rates of respiration than wild-type controls. We also noticed that the marked reduction in respiratory activity (0.45 μM/s) of Δ*cydAB Salmonella* treated with NO for 1 min was sustained for the duration of the experiment. Nonetheless, the respiratory activity of Δ*cydAB Salmonella* recovered to the levels of untreated specimens 10 min after the addition of NO (0.70 and 0.69 μM O_2_/s for untreated and NO-treated samples, respectively), suggesting that *Salmonella* can eventually adapt to nitrosative stress in the absence of cytochrome *bd*. It should be noted that the respiratory activity of Δ*cydAB Salmonella* decreased after 10 min of culture, likely reflecting reduced affinity of cytochrome *bo* for O_2_ as bacterial density increases over time.

Because the Δ*hmp* mutant is highly susceptible to the antimicrobial activity of NO (Fig. 2) and because Hmp is a critical antinitrosative defense that promotes respiratory activity in Gram-negative bacteria undergoing nitrosative stress (37), we also examined the effects of NO on respiration of Δ*hmp Salmonella* (Fig. 3C). Compared to Δ*cydAB* isogenic controls, respiration of Δ*hmp Salmonella* was more profoundly inhibited 1 min after exposure to 50 μM spermine NONOate (0.45 versus 0.23 μM O_2_/s, respectively), suggesting that Hmp is more efficient at detoxifying NO than cytochrome *bd*. Interestingly, Δ*hmp Salmonella* recovered more than 50% respiratory activity 10 min after NO treatment (1.70 versus 0.96 μM O_2_/s in untreated and NO-treated specimens, respectively), indicating the existence of Hmp-independent means to detoxify NO.

We finally quantified the O_2_-consuming capacity of a mutant lacking both *hmp* and *cydAB*. The Δ*hmp* Δ*cydAB*::Km *Salmonella* strain AV09592 suffered as much repression of O_2_ consumption as its Δ*hmp* isogenic control 1 min after the addition of 50 μM spermine NONOate (0.20 versus 0.23 μM O_2_/s, respectively). However, compared to Δ*hmp* controls, the respiration of Δ*hmp* Δ*cydAB*::Km *Salmonella* remained more profoundly inhibited 10 min after NO treatment (1.48 versus 0.44 μM O_2_/s). Together, these data indicate that cytochrome *bd* adds to the dominant NO-detoxifying activity of the flavohemoglobin Hmp. Because the Δ*hmp* Δ*cydAB* mutant partially recovered its respiratory activity 10 min after NO treatment, these investigations also point to the existence of Hmp- and cytochrome *bd*-independent means to detoxify NO.

### Contribution of Hmp and cytochrome *bd* to *Salmonella* pathogenesis.

**FIG 4:**
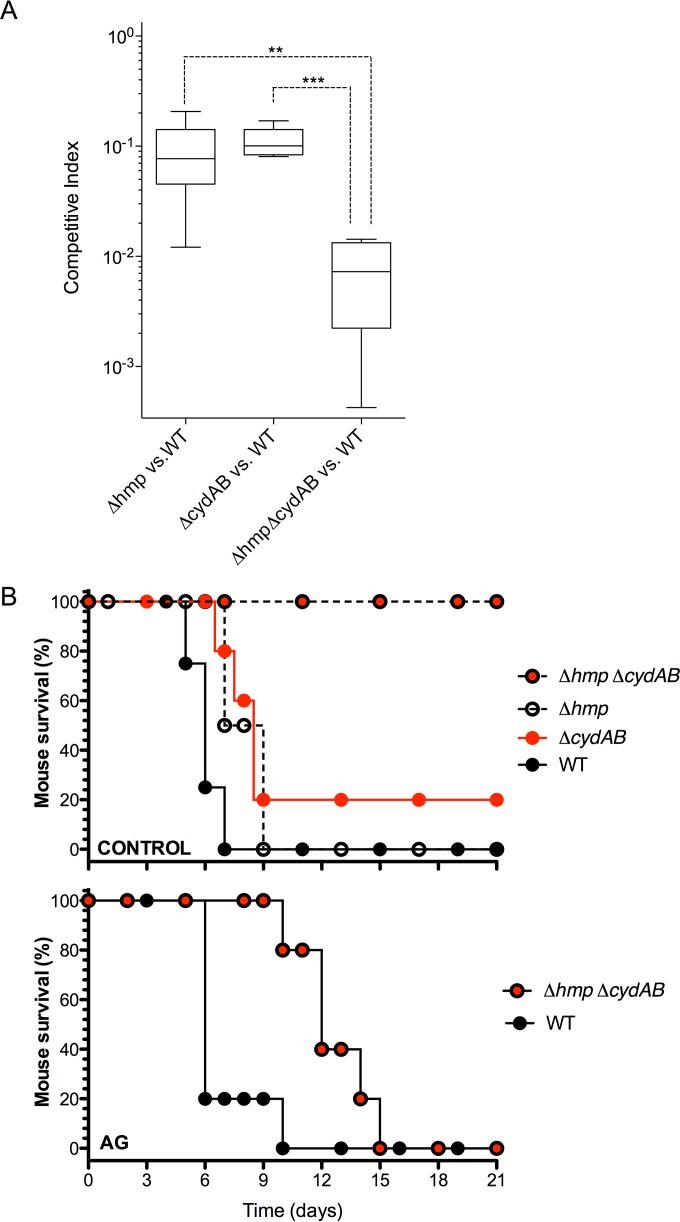
Hmp and cytochrome *bd* in *Salmonella* pathogenesis. (A) The competitive index was measured in livers of C3H/HeN mice 3 days after i.p. inoculation with 2,000 CFU of a mixture containing wild-type (WT) and equal numbers of Δ*cydAB*::Km, Δ*hmp*::Km, and Δ*hmp* Δ*cydAB*::Km *Salmonella*. **, *P* < 0.01; ***, *P* < 0.001. (B) Survival of *Salmonella*-infected C3H/HeN mice was recorded over time after i.p. inoculation. Selected groups of mice were continuously fed water containing 500 μg/ml of the iNOS inhibitor aminoguanidine (AG). The Δ*cydAB* Δ*hmp* mutant strain was found to be attenuated (*P* < 0.0001) in C3H/HeN mice according to the log rank Mantel-Cox survival test. The data are from 5 to 7 mice per group.

Having established that both Hmp and cytochrome *bd* protect respiration of *Salmonella* experiencing nitrosative stress, we used two murine models to examine the extent to which these two antinitrosative defenses contribute to *Salmonella* pathogenesis. First, C3H/HeN mice were inoculated intraperitoneally (i.p.) with equal numbers of wild-type and Δ*hmp*, Δ*cydAB*, or Δ*hmp* Δ*cydAB*::Km *Salmonella*, and the competitive advantage of bacteria in the mixtures was determined by quantifying hepatic burden 5 days after infection. These investigations showed that Δ*hmp* and Δ*cydAB Salmonella* are similarly attenuated (competitive indexes of about 0.1 and *P* > 0.05 compared to wild-type *Salmonella*) (Fig. 4A). In contrast, Δ*hmp* Δ*cydAB*::Km *Salmonella* had a competitive index of 0.01 compared to wild-type bacteria. The double mutant was found to be significantly more attenuated than either Δ*hmp* (*P* < 0.01) or Δ*cydAB* (*P* < 0.001) *Salmonella*. Together, these findings suggest a substantial degree of independence between Hmp and cytochrome *bd* in *Salmonella* pathogenesis. We also examined the relative contributions of Hmp and cytochrome *bd* in a live/dead model of acute *Salmonella* infection (Fig. 4B). Strain AV09592 harboring mutations in both *hmp* and *cydAB* was significantly (*P* < 0.0001) more attenuated than wild-type, Δ*hmp*, or Δ*cydAB Salmonella*. Administration of the inducible nitric oxide synthase (iNOS) inhibitor aminoguanidine increased the virulence of Δ*hmp* Δ*cydAB*::Km *Salmonella*. Aminoguanidine-treated, Δ*hmp* Δ*cydAB*::Km *Salmonella-*infected mice died a few days after controls challenged with wild-type bacteria. Collectively, our investigations indicate that Hmp and cytochrome *bd* are important components of the antinitrosative toolbox of *Salmonella*.

## DISCUSSION

*Salmonella* must adapt to various concentrations of O_2_ and NO in different anatomical sites during the course of an infection. Differential utilization of quinol oxidases with distinct affinities for O_2_ and NO allows *Salmonella* to colonize the gastrointestinal tract and to establish infections in deep tissue (this study and reference 28). Our investigations have demonstrated that the ability of cytochrome *bd* to protect the electron transport chain against NO is a considerable component of the adaptive antinitrosative defenses of *Salmonella* in a murine model of acute systemic infection. The high affinity for NO of cytochrome *bd* (*K*_*d*_ [dissociation constant] of 0.55 nM) provides a molecular mechanism by which this quinol oxidase contributes to antinitrosative defenses (36). Preferential nitrosylation of cytochrome *bd* frees up cytochrome *bo* for respiration. In addition to having high affinity for NO, cytochrome *bd* dissociates faster from NO than most known cytochromes, including cytochrome *bo* (36). The fast dissociation of NO from heme *d* may explain why we noted that Δ*cyoABCD Salmonella* expressing cytochrome *bd* maintains excellent respiratory activity in the presence of NO. Compared to cytochrome *bo*, fast dissociation of NO from heme *d* may also underlie the lower sensitivity of cytochrome *bd* to this diatomic radical. Cytochrome *bd* is resistant not just to NO but also to hydrogen sulfide (38, 39). Expression of cytochrome *bd* may therefore preserve respiratory activity in hypoxic environments in the presence of sulfide and NO.

Cytochrome *bd* may mediate antinitrosative defense through nitrosyl and nitrate pathways. According to the nitrosyl pathway, ferrous iron in heme *d* binds NO in competition with O_2_. The nitrosyl (Fe*_d_*^2+^-NO) product resulting from this reaction dissociates with a *K*_off_ of 0.133 s^−1^ (40). The nitrosyl pathway prevails at high e^−^ flux and low O_2_ concentrations, conditions that appear to be encountered by *Salmonella* in hypoxic systemic tissues (29). Second, according to the nitrite pathway, the oxoferryl (Fe*_d_*^4+^=O) intermediate can react with NO with a second-order rate constant of 10^5^ M^−1^ s^−1^ (41). The NO_2_^−^ anion formed at the catalytic site is ejected successfully from the Fe*_d_*^3+^-NO_2_^−^ intermediate, providing a direct mechanism for NO detoxification. The nitrite pathway, which proceeds under low e^−^ flux and high O_2_ conditions, provides a rationale for the noncompetitive inhibition of cytochrome *bd* at low NO/O_2_ ratios. Because the flavohemoglobin Hmp, whose enzymatic activity operates at high O_2_ concentrations (42), plays a role in *Salmonella* pathogenesis (22), *Salmonella* is likely to encounter host-derived NO under high O_2_ tensions. Therefore, it is possible that cytochrome *bd* detoxifies NO *in vivo* by the nitrite pathway. Whether cytochrome *bd* uses the nitrosyl or nitrite pathways depends on changing NO/O_2_ ratios during the course of the infection.

*Salmonella* encounters NO and its congeners in the gastrointestinal tract and within mononuclear phagocytic cells (8, 9, 43). *Salmonella* is remarkably resistant to the antimicrobial activity of NO generated in the innate host response (8). Reaction of reactive nitrogen species with low-molecular-weight thiols such as homocysteine and glutathione contributes to *Salmonella* pathogenesis (20, 21). *Salmonella* can also detoxify NO to nitrous oxide (N_2_O) or NO_3_^−^ through the enzymatic activities of the anaerobic flavorubredoxin NorVW or the aerobic flavohemoglobin Hmp, respectively (22, 44, 45). Acute murine models of infection have shown that Hmp, not NorVW, defends *Salmonella* against nitrosative stress generated in the host (22), suggesting that aerobic conversion of NO to NO_3_^−^ is a biologically relevant pathway for detoxification of host-derived NO. Our investigations indicate that, together with Hmp, cytochrome *bd* is an important component of the adaptive antinitrosative arsenal of *Salmonella in vivo*. Our biochemical and microbiological approaches suggest that Hmp is more important than cytochrome *bd* in protecting the respiratory and replicative capacity of *Salmonella* exposed to chemically generated NO. The competitive assays recorded in mice indicate, however, that both Hmp and cytochrome *bd* contribute to similar extents to *Salmonella* pathogenesis. Several reasons may explain the ranking of importance for Hmp and cytochrome *bd* as antinitrosative defenses of *Salmonella* depending on the experimental conditions tested. First, limited O_2_ concentrations in mice might favor NO detoxification by cytochrome *bd*, whereas high P_O2_ tension in culture may favor Hmp enzymatic activity. Second, *Salmonella* may experience different O_2_ and NO concentrations as the inflammatory response evolves over time in the course of the infection. Thereby, *Salmonella* may preferentially use Hmp or cytochrome *bd* according to the availability of O_2_ and NO. Unique utilization of Hmp and cytochrome *bd* could explain why Δ*hmp* Δ*cydAB*::Km *Salmonella* is significantly more attenuated than mutants lacking *hmp* or *cydAB*. Third, it is also possible that Hmp and cytochrome *bd* may perform redundant NO detoxification at high O_2_ tensions. Thus, the absence of both antinitrosative defenses in Δ*hmp* Δ*cydAB*::Km *Salmonella* accentuates susceptibility to NO.

Cytochrome *bd* may also add to *Salmonella* pathogenesis in ways that are independent of NO detoxification. The electrogenic quinol-O_2_ oxidoreductase activity of cytochrome *bd* generates a proton motive force across the membrane that fuels oxidative phosphorylation (46). The importance of energetics in *Salmonella* pathogenesis is suggested by the observation that inhibition of iNOS did not completely restore virulence of Δ*hmp* Δ*cydAB*::Km *Salmonella*. Moreover, our biochemical and microbiological analyses showed that Δ*cydAB Salmonella* has lower rates of respiration and growth than wild-type or Δ*cyoABCD* controls. The high affinity of cytochrome *bd* for O_2_ could allow *Salmonella* to colonize hypoxic areas in the host. In this sense, cytochrome *bd* promotes growth of *Salmonella* in systemic sites but seems to play a marginal role in colonization of gut mucosa (this work and references 28 and 30). These patterns likely reflect the fact that cytochrome *bd* works at a 5 to 10% O_2_ tension in tissue but performs poorly at 0.8% O_2_ in gut lumen (28). In addition to canonical energetic functions, the high affinity of cytochrome *bd* for O_2_ could protect vulnerable [4Fe-4S] clusters in dehydratases and transcription factors such as fumarate-nitrate reduction regulator (FNR) from oxidative damage. Finally, cytochrome *bd* is an important source of oxidizing power that aids the DsbA-DsbB-ubiquinone complex with the formation of disulfide bonds and folding of periplasmic proteins (47). In this fashion, cytochrome *bd* could fuel DsbA-dependent folding of components of the *Salmonella* pathogenicity island 2 type III secretion system (48), a nanomachine that is essential for the intracellular replication of *Salmonella* as well as resistance of this facultative intracellular pathogen to oxygen-dependent and -independent antimicrobial host defenses.

**FIG 5:**
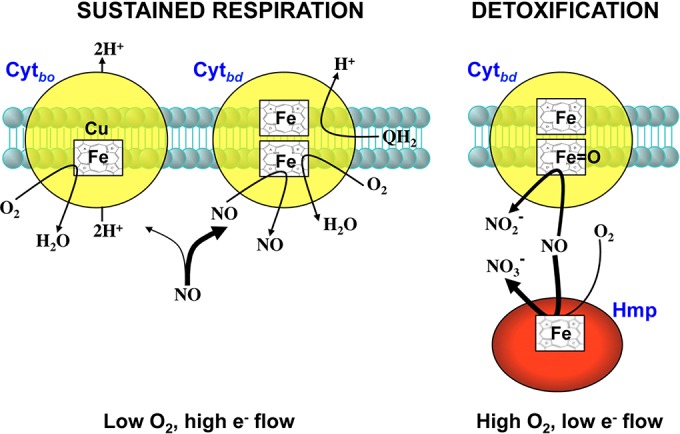
Bioenergetic and antinitrosative roles of cytochrome *bd* in *Salmonella* pathogenesis. Cytochrome *bd* promotes antinitrosative defenses by ensuring respiratory activity in the presence of NO (left) and by detoxifying NO (right). At high NO/O_2_ ratios and high electron flow, the high affinity of cytochrome *bd* for NO ensures that cytochrome *bo* is free to respire in *Salmonella* undergoing nitrosative stress. In addition, the high *K*_off_ of ferrous iron in heme *d* for NO allows cytochrome *bd* to reduce O_2_ to water. 2H^+^ and 1H^+^ are translocated by the actions of cytochrome *bo* and cytochrome *bd*, respectively. At low NO/O_2_ ratios and low electron flow, NO reacts with the oxoferryl intermediate in heme_*d*_ of cytochrome *bd*, yielding the oxidative product nitrite (NO_2_^−^). The dominant denitrosylase enzymatic activity of the flavohemoglobin Hmp detoxifies NO to NO_3_^−^. Synergism between cytochrome *bd* and Hmp potentiates antinitrosative defenses of *Salmonella*. The width of the arrows represents, in this order, the hierarchical binding of NO to Hmp, cytochrome *bd*, and cytochrome *bo*.

In summary, our investigations indicate that dual functions of cytochrome *bd* in bacterial bioenergetics and antinitrosative defenses contribute to *Salmonella* pathogenesis in murine models of systemic infection (Fig. 5). Generation of electrochemical gradients across cytoplasmic membranes, oxidation and folding of periplasmic proteins, and detoxification of O_2_ and NO represent some of the diverse mechanisms by which cytochrome *bd* may promote bacterial growth in inflammatory and normal tissue hypoxia.

## MATERIALS AND METHODS

### Bacterial strains.

Strains and primers used in these investigations are listed in Tables S1 and S2 in the supplemental material. Mutations were constructed following the λ Red recombinase system (49). In-frame deletions were verified by PCR analysis.

### Susceptibility to NO.

The effects of the polyamine diethylenetriamine (DETA) or the NO donor DETA NONOate on growth of wild-type and mutant *Salmonella* were measured spectrometrically on a Bioscreen C microbiology microtiter plate (Growth Curves USA, Piscataway, NJ). *Salmonella* cultures grown overnight in LB broth were diluted 1:500 in LB broth and treated with 5 mM NO donor DETA NONOate or the polyamine DETA control. Where indicated, some cultures were independently treated with 1 mM DETA NONOate. The half-life (*t*_1/2_) of DETA NONOate at neutral pH is about 20 h. We estimate that 5 mM DETA NONOate produced a rather stable flux of 5 μM NO for 20 h that lasted the experiment (50). Bacterial growth was recorded as optical density at 600 nm (OD_600_) every 15 min, while cultures were shaken at 37°C.

### Cytochrome spectrometry.

Wild-type and mutant *Salmonella* strains grown overnight in LB broth were subcultured in LB broth to an OD_600_ of 0.5. Inner membranes were prepared as described by Husain et al. (11). Briefly, bacterial pellets were resuspended in 10 mM EDTA, 100 mM Tris-HCl buffer, pH 8.5. Bacteria were lysed by passing the cell suspension through a French press cell disruptor (Thermo Electron Corporation, Milford, MA) 3 times at 18,000 lb/in^2^ at a flow rate of 5 ml/min. Cell debris was removed after centrifugation at 10,000 × *g* for 20 min. The supernatants were then centrifuged at 200,000 × *g* for 1 h, and the pellets were solubilized in 75 mM K_2_HPO_4_, 150 mM KCl, 5 mM EDTA, 60 mM *N*-dodecyl-*N*,*N*-dimethyl-3-ammonio-1-propanesulfonate buffer, pH 6.4. Supernatants containing inner membranes were collected, and the protein concentration was assayed using the bicinchoninic acid (BCA) protein assay kit (Thermo Fisher Scientific, Rockford, IL). The protein concentration in the specimens was adjusted to 1.5 mg/ml in 75 mM K_2_HPO_4_, 150 mM KCl, 5 mM EDTA, 10 mM ascorbate, and 60 mM *N*-dodecyl-*N*,*N*-dimethyl-3-ammonio-1-propanesulfonate buffer, pH 6.4. Absorbance spectroscopy was collected in a Cary 50 Bio UV-visible spectrophotometer. Cytochrome content also was evaluated by difference spectroscopy. Briefly, stationary-phase wild-type, Δ*hmp,* and Δ*cydAB Salmonella* strains grown overnight in LB broth were subcultured 1:100 in EG medium (E salts [1.66 mM MgSO_4_, 9.5 mM citric acid monohydrate, 57 mM K_2_HPO_4_, 16.7 mM NaNH_3_PO_4_] supplemented with 0.4% [wt/vol] glucose), pH 7.0, for 4 h. Bacterial densities were adjusted to an OD_600_ of 0.1. Some of the specimens were oxidized with 10 mM ammonium persulfate for 10 min before measuring cytochrome content by UV-visible spectroscopy. Cytochrome content in bacterial cells was estimated by recording reduced-minus-oxidized spectra.

### Transcriptional analysis.

Wild-type, Δ*hmp*, or Δ*cydAB Salmonella* strains grown overnight in LB broth were subcultured 1:100 in LB broth and grown at 37°C with shaking to an OD_600_ of 0.5. The cultures were treated with 50 μM spermine NONOate for 3, 5, or 15 min at 37°C with shaking. Cultures were then combined 5:1 with a mixture of ice-cold phenol (5%)–ethanol (95%), incubated on ice for 10 min, and pelleted by centrifugation. RNA isolation was performed using the High Pure RNA isolation kit (Roche, Basel, Switzerland) according to the manufacturer’s instructions for bacterial samples and included on-column DNase treatment. cDNA was prepared from 1 μg total RNA using 0.45 μM N6 random hexamer primers (Life Technologies, Carlsbad, CA) and 100 U of Moloney murine leukemia virus (MMLV) reverse transcriptase (Promega, Madison, WI). The primers and probes used for quantitative PCR (qPCR) are listed in Table S3 in the supplemental material. Reaction mixtures were prepared using TaqMan Gene Expression Master Mix (Life Technologies) and were incubated at 50°C for 2 min and then 95°C for 10 min, prior to 40 cycles of 95°C for 15 s and 57°C for 1 min. The expression of *cydA* was normalized to the expression of the *rpoD* housekeeping gene.

### O_2_ measurements.

*Salmonella* grown overnight in LB broth was diluted 1:100 in EG medium. Bacteria were grown at 37°C in a shaker incubator until they reached an OD_600_ of 0.5. The cultures were diluted to an OD_600_ of 0.2 in EG medium and equilibrated in a shaker incubator at 37°C for 3 min before they were transferred into an air-sealed, multiport measurement chamber equipped with an Iso-Oxy-2 O_2_ probe. The evolution of O_2_ in the cultures was recorded with an Apollo 4000 free radical analyzer (World Precision Instruments, Inc., Sarasota, FL). To assess the ability of the bacteria to adapt to NO, O_2_ consumption was also studied in cultures treated for 10 min with 50 μM spermine NONOate (*t*_1/2_ = 39 min at 37°C). The data are expressed as micromolar concentrations of O_2_.

### Bacterial virulence in mice.

Eight- to 10-week-old NRAMP1^R^ C3H/HeN mice were bred at the animal facility of the University of Colorado School of Medicine according to Institutional Animal Care and Use Committee guidelines. C3H/HeN mice were treated with 20 mg/mouse 1 day before intragastric infection with 10^8^ CFU of *Salmonella* prepared in phosphate-buffered saline (PBS) from overnight cultures grown in LB broth. *Salmonella* shedding was examined over time in fecal pellets. The abilities of wild-type and mutant *Salmonella* to colonize ileum, cecum, and colon were measured 5 days after infection. C3H/HeN mice were independently inoculated i.p. with ~2,000 CFU of a bacterial mixture containing equal numbers of wild-type and mutant *Salmonella* (51). After 3 days of infection, the bacterial burden in livers was quantified on LB agar plates containing the appropriate antibiotics. The competitive index was calculated according to the formula (strain 1/strain 2)_output_/(strain 1/strain 2)_input_. In addition, C3H/HeN mice were inoculated intraperitoneally with 1 × 10^3^ to 3 × 10^3^ CFU/mouse of wild-type or mutant *Salmonella*. Where indicated, the drinking water of selected groups of C3H/HeN mice was supplemented with 500 μg/ml of the iNOS-specific inhibitor aminoguanidine. The survival of *Salmonella*-infected mice was recorded over time.

### Histopathology.

Ceca were scored for submucosal edema, neutrophil infiltration into the lamina propria, and goblet cell number per 400× field as described previously (52). Ten fields per animal per tissue were examined. Images demonstrating representative fields were captured on an Olympus BX51 microscope equipped with a 4-megapixel Macrofire digital camera (Optronics, Goleta, CA) using the PictureFrame application 2.3 (Optronics). Composite images were assembled with the use of Adobe Photoshop. All images in the composite were handled identically.

### Statistical analysis.

One-way analysis of variance, followed by a Bonferroni posttest, was used to establish statistical significance. Differences in mouse survival of *Salmonella* infection were determined by a log rank Mantel-Cox test. A *P* value of <0.05 was considered significant.

## SUPPLEMENTAL MATERIAL

Table S1  Bacterial strains. Table S1, DOCX file, 0.1 MB

Table S2  Primers. Table S2, DOCX file, 0.04 MB

Table S3  Primers and probes for qPCR. Table S3, DOCX file, 0.04 MB
