# Supplementary material for: Cytochrome bd-Dependent Bioenergetics and Antinitrosative Defenses in Salmonella Pathogenesis
Source: mBio. 2016 Dec 20;7(6):e02052-16. doi: 10.1128/mBio.02052-16 (PMC5181779; doi:10.1128/mBio.02052-16)
Supplement: Table S1 — Bacterial strains. [file mbo006163115st1.docx]

Supplementary tables.

Table S1. Bacterial strains.

| Strain or Plasmid | Relevant characteristic | Source or Reference |
| --- | --- | --- |
| Strains |  |  |
| *S.* Typhimurium strain 14028s | Wild-type | ATCC |
| AV0468 | *Δhmp*::FRT | (1) |
| AV0429 | *ΔcyoABCD::*FRT | (2) |
| AV0543 | *ΔcydAB::*FRT | This study |
| AV09592 | *Δhmp::*FRT *ΔcydAB::km* | This study |
| Plasmids |  |  |
| pKD13 | *bla* FRT *ahp* FRT *PS1 PS4 ori*R6K | (3) |
| pCP20 | *bla cat c*I857 *λ*P_R_ *flp* pSC101 oriTS | (4) |
|  |  |  |

Table S2. Primers**.**

| Mutation constructed | Primer Sequence |
| --- | --- |
| *ΔcydAB::km* | F:5’-GGGTGTGGCTACCGGTTTGACCATGGAGTTCCAGTTCGGGACAAACTGGTCGTACTACTCGCTGGAGCTGCTTCGAAGTT |
|  | R:5’-AACAGGAATGCCCACGCGCCTTTCTCCATACGGGAAGTCAGGATAGTCAGCAGCGGCAGATTCCGGGGATCCGTCGACCT |
| Mutation confirmation | Primer Sequence |
| *cydAB* | F:5’-TAGTCGAACTGTCGCGCTTA |
|  | R:5’-ACCCAGGTCATCAGGTTCAG |

Table S3. Primers and probes for qPCR**.**

| *cydA* | F:5’-TTCTTCGGCTGGGATCGTCT |
| --- | --- |
|  | R:5’-GAGAAGCTCACCATTTCCATACGC |
|  | Probe: 6-FAM-CAGCCGTTCGCTACCAGAATCCACA-BHQ1 |
| *rpoD* | F:5’-GTGGCTTGCAATTCCTTGAT |
|  | R:5’-AGCATCTGGCGAGAAATACG |
|  | Probe: 6-FAM-ATAAGTTCGAATACCGTCGCGGCTACA-BHQ1 |

**References:**

**1. McCollister BD, Bourret TJ, Gill R, Jones-Carson J, V·zquez-Torres A.** 2005. Repression of SPI2 transcription by nitric oxide-producing, IFNγ-activated macrophages promotes maturation of *Salmonella* phagosomes. J. Exp. Med. **202:**625

**2. Husain M, Bourret TJ, McCollister BD, Jones-Carson J, Laughlin J, Vazquez-Torres A.** 2008. Nitric oxide evokes an adaptive response to oxidative stress by arresting respiration. J Biol Chem **283:**7682-7689.

**3. Datsenko KA, Wanner BL.** 2000. One-step inactivation of chromosomal genes in *Escherichia coli* K-12 using PCR products. Proc Natl Acad Sci U S A **97:**6640-6645.

**4. Cherepanov PP, Wackernagel W.** 1995. Gene disruption in *Escherichia coli*: Tc^R^ and Km^R^ cassettes with the option of Flp-catalyzed excision of the antibiotic-resistance determinant. Gene **158:**9-14.
