# Supplementary material for: Cytochrome bd-Dependent Bioenergetics and Antinitrosative Defenses in Salmonella Pathogenesis
Source: mBio. 2016 Dec 20;7(6):e02052-16. doi: 10.1128/mBio.02052-16 (PMC5181779; doi:10.1128/mBio.02052-16)
Supplement: Table S2 — Primers. [file mbo006163115st2.docx]

Table S2. Primers**.**

| Mutation constructed | Primer Sequence |
| --- | --- |
| *ΔcydAB::km* | F:5’-GGGTGTGGCTACCGGTTTGACCATGGAGTTCCAGTTCGGGACAAACTGGTCGTACTACTCGCTGGAGCTGCTTCGAAGTT |
|  | R:5’-AACAGGAATGCCCACGCGCCTTTCTCCATACGGGAAGTCAGGATAGTCAGCAGCGGCAGATTCCGGGGATCCGTCGACCT |
| Mutation confirmation | Primer Sequence |
| *cydAB* | F:5’-TAGTCGAACTGTCGCGCTTA |
|  | R:5’-ACCCAGGTCATCAGGTTCAG |
