# Supplementary material for: Cytochrome bd-Dependent Bioenergetics and Antinitrosative Defenses in Salmonella Pathogenesis
Source: mBio. 2016 Dec 20;7(6):e02052-16. doi: 10.1128/mBio.02052-16 (PMC5181779; doi:10.1128/mBio.02052-16)
Supplement: Table S3 — Primers and probes for qPCR. [file mbo006163115st3.docx]

Table S3. Primers and probes for qPCR**.**

| *cydA* | F:5’-TTCTTCGGCTGGGATCGTCT |
| --- | --- |
|  | R:5’-GAGAAGCTCACCATTTCCATACGC |
|  | Probe: 6-FAM-CAGCCGTTCGCTACCAGAATCCACA-BHQ1 |
| *rpoD* | F:5’-GTGGCTTGCAATTCCTTGAT |
|  | R:5’-AGCATCTGGCGAGAAATACG |
|  | Probe: 6-FAM-ATAAGTTCGAATACCGTCGCGGCTACA-BHQ1 |
